# Adrenal Insufficiency in Patients with Beta Thalassemia: A Meta-Analysis

**DOI:** 10.3390/medicina60101571

**Published:** 2024-09-25

**Authors:** Christos Savvidis, Dimitra Ragia, Sophia Delicou, Aikaterini Xydaki, Manfredi Rizzo, Ioannis Ilias

**Affiliations:** 1 Department of Endocrinology, Hippocration General Hospital, GR-11527 Athens, Greece; csendo@yahoo.gr (C.S.); dimitraragia@gmail.com (D.R.); 2 Thalassemia and Sickle Cell Unit, Expertise Center of Hemoglobinopathies and Their Complications, Hippocration General Hospital, GR-11527 Athens, Greece; sophiadelicou@gmail.com (S.D.); katerina.xydaki@gmail.com (A.X.); 3 School of Medicine, Department of Health Promotion, Mother and Child Care, Internal Medicine and Medical Specialties (Promise), University of Palermo, 90133 Palermo, Italy; manfredi.rizzo@unipa.it

**Keywords:** adrenal insufficiency, thalassemia, transfusion-dependent anemias, meta-analysis, prevalence

## Abstract

*Background and Objectives*: Adrenal insufficiency (AI) can be a significant concern in patients with transfusion-dependent homozygous beta thalassemia (bThal) due to the chronic disease burden and frequent blood transfusions that these patients require. The prevalence of AI in this population remains unclear, with studies often lacking control groups for comparison. This meta-analysis aimed to estimate the proportion of patients with transfusion-dependent bThal who exhibit evidence of AI. *Materials and Methods*: A systematic review following PRISMA guidelines identified 19 studies for analysis. *Results*: Despite the variability in the diagnostic methods used to ascertain AI, the meta-analysis revealed that approximately one-third of patients had evidence of AI, with the prevalence rising to 50% in studies focused on adults with bThal. *Conclusions*: These findings suggest an increased risk of AI in patients with bThal compared to the general population. Clinicians should consider tailored management strategies, including glucocorticoid coverage during surgical procedures, to mitigate the risk of adrenal crises in this vulnerable patient group. Further research is needed to optimize adrenal surveillance and management in patients with bThal.

## 1. Introduction

Homozygous beta thalassemia (bThal) can significantly impact endocrine function, primarily due to chronic iron overload resulting from regular blood transfusions. The inability of the body to excrete excess iron leads to iron accumulating in various organs, including endocrine glands such as the pituitary, thyroid, parathyroid, pancreas, and gonads. Iron deposition in these glands causes oxidative stress and cellular damage, disrupting hormone secretion and regulation. Chelation therapy is essential for managing iron overload and protecting endocrine organs, but the chelating agents themselves may influence endocrine function (this may result from the chelation of other essential trace elements like zinc when iron levels are low, the direct toxic effects of unchelated deferoxamine through interference with vital iron-dependent enzymes, or a combination of both factors) [1]. The hypothalamic–pituitary–adrenal (HPA) axis is a central component of the body’s neuroendocrine system, responsible for regulating stress responses, mood, immune function, and energy metabolism. The hypothalamus secretes corticotropin-releasing hormone (CRH) in response to stress. CRH stimulates the anterior pituitary gland to release adrenocorticotropic hormone (ACTH), which then signals the adrenal cortex to produce and release glucocorticoids, primarily cortisol. Cortisol exerts widespread effects, including maintaining blood pressure, regulating glucose metabolism, and modulating the immune response. The HPA axis operates through a negative feedback loop, where elevated cortisol levels inhibit further CRH and ACTH release, thus maintaining hormonal balance and stress adaptation. Disruptions in the HPA axis can contribute to various clinical disorders, including adrenal insufficiency (AI) [2]. Adrenal insufficiency (AI) is a potentially life-threatening condition characterized by inadequate production of glucocorticoids, mineralocorticoids, or both. The reported prevalence of AI in the general population is approximately 8–14 per 100,000 individuals (primary AI) and 15–28 per 100,000 individuals (secondary or central AI) [3]. The prevalence of AI in patients with bThal remains unclear [4,5]. These patients receive multiple blood transfusions and iron chelation therapy, which may affect the HPA axis. Moreover, the methods used to diagnose AI in this population vary across studies, with some relying on baseline laboratory measurements, and others using dynamic testing.

The association between adrenal dysfunction and transfusion-dependent anemias has generated interest among clinicians and researchers [6]. However, most studies in this area lack appropriate control groups, hindering definitive assessments of prevalence and risk factors. This meta-analysis aimed to estimate the proportion of patients with bThal who exhibit evidence of AI, taking into consideration when the available studies were conducted, the age of the studied subjects, and the various diagnostic means to evaluate AI.

## 2. Materials and Methods

**Figure 1:**
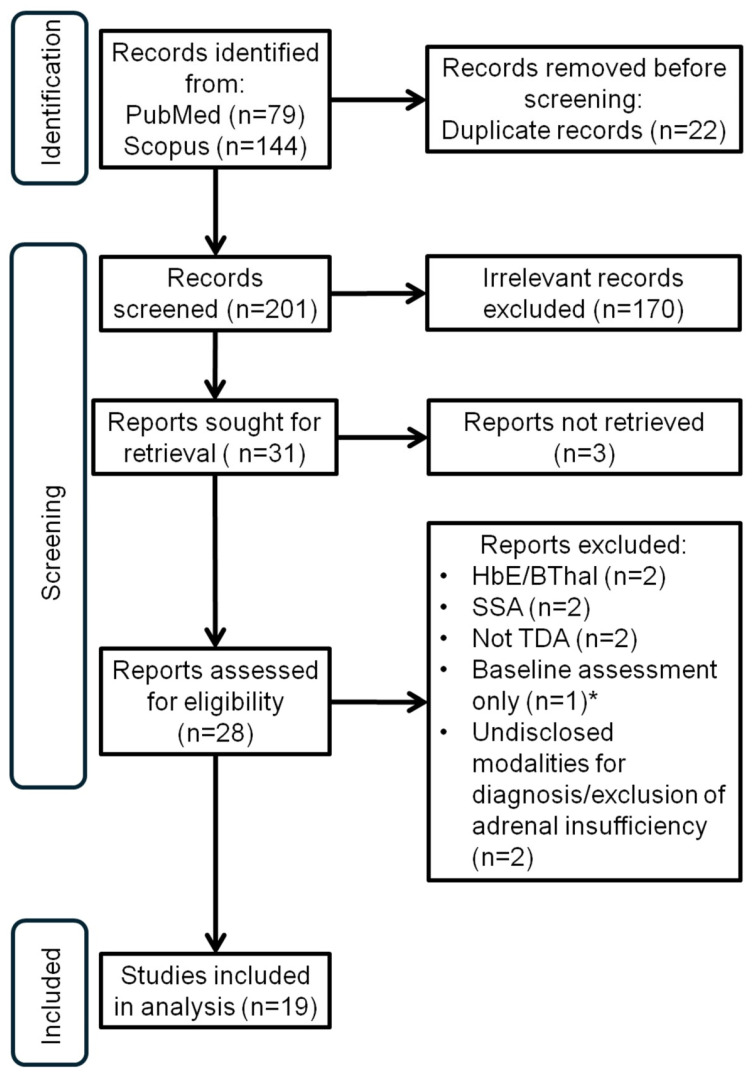
PRISMA diagram for the study; HbE/Bthal (hemoglobin E-beta thalassaemia); SSA (sickle-cell anemia); TDA (transfusion-dependent anemia); * baseline assessment of adrenal reserve, with results in a “grey” zone, but considered by the authors as being normal (see Section 4).

A systematic literature review [PROSPERO registration No: CRD42024536719; [https://www.crd.york.ac.uk/prospero/display_record.php?RecordID=536719, accessed on 21 September 2024] was conducted following PRISMA guidelines. PubMed and Scopus databases were searched using the strategy “human AND ((adrenal AND insufficiency) OR (adrenal AND failure)) AND (thalassemia AND (transfusion AND dependent AND anemia))”. Initially, 79 articles were identified from PubMed and 144 from Scopus. After screening and removal of works examining other forms of transfusion-dependent anemias or sickle cell anemias, works where the evaluation for adrenal insufficiency was conducted with baseline-only testing or undisclosed modalities, as well as duplicate publications, 19 articles were included for meta-analysis (see the PRISMA diagram—Figure 1) [6,7,8,9,10,11,12,13,14,15,16,17,18,19,20,21,22,23,24,25,26,27].

The quality of the articles was assessed with the University of Pennsylvania modified version of the Newcastle–Ottawa scale (NOS) (https://www.med.upenn.edu/CEP/assets/user-content/documents/modified-newcastle-ottawa.pdf, accessed on 21 September 2024) (Supplemental Table S1); all the studies had a score of 4/10 (a major limitation being the absence of control subjects). Given the absence of control groups, a meta-analysis of proportions was performed. Analysis was performed for all the data combined according to AI testing modality, age of the included subjects, and year of publication.

## 3. Results

Six hundred and sixty-three subjects were included in the meta-analysis. Most studies were conducted in young persons < 24 y.o. and implemented dynamic testing with tetracosactrin (synthetic ACTH administration); few studies performed other dynamic tests (glucagon stimulation testing (GST) or insulin tolerance testing (ITT)) (Table 1).

**Figure 2:**
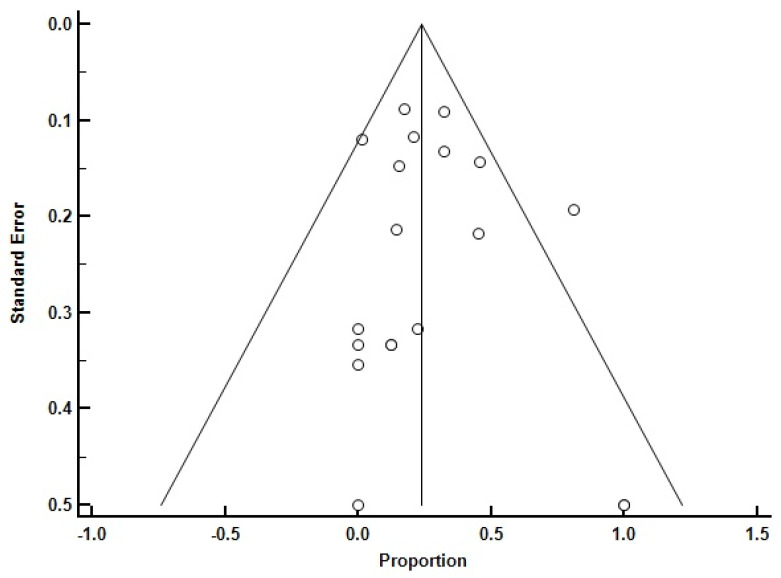
Funnel plot of all the studies which were included in the analysis (Q: 136.86; *p* < 0.0001; I^2^: 86.85%; 95% CI for I^2^: 80.88 to 90.95).

Some studies that established their reported results with baseline-only testing orundisclosed/unspecified modalities were excluded from the analysis (see previous section). Some indication of bias was seen in funnel plots; heterogeneity indexes ranged from 6.70 to 136.86 (Q) and 25.48% to 94.55% (I2); and no heterogeneity was seen in studies focused only on adults (however, there were fewer than 10 such studies) (Figure 2 and Supplemental Figure S1).

**Figure 3:**
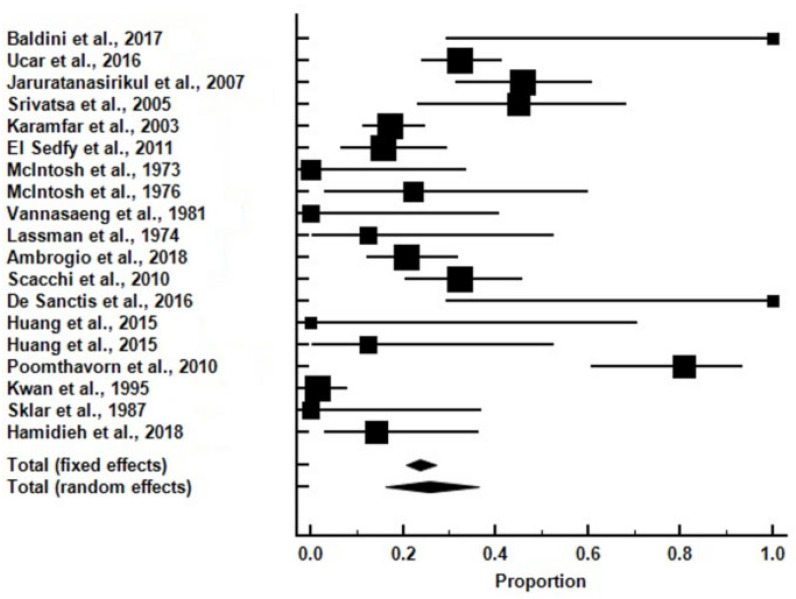
Forrest plot of all the studies which were included in the analysis (percentage of adrenal insufficiency: 23.81% [fixed effects] and 25.62% [random effects]) [6,7,8,9,10,11,12,13,15,16,17,18,19,20,21,22,23,27].

The meta-analysis of the 19 selected studies revealed that a substantial percentage of patients with transfusion-dependent bThal had evidence of AI, with the prevalence ranging from 0% to 80% across individual studies (Figure 3 and Supplemental Figure S2).

Overall, one-quarter of all the subjects with bThal had AI (25.62%). Specifically, in subjects who were assessed with tetracosactrin (Synacthen; either low-dose [post1 μgtetracosactrini.v.] and/or high-dose [post250 μgtetracosactrini.v.]), AI was found in 27.25%, whereas the rate of AI was lower at 17.17% in subjects who were assessed with ITT. The highest rate of AI was noted in adults > 24 y.o. (51.5%) compared to those < 24 y.o. who had a rate of 20.34%. In studies conducted before the year 2000, the rate was low at 5.35%, in sharp contrast to those conducted after the year 2000, where the rate increased to 35.36%.

## 4. Discussion

AI in bThal results from iron deposition/secondary hemochromatosis in the adrenal cortex (primary AI) or the pituitary gland (secondary or central AI; the latter term usually also includes cases of hypothalamic causes of AI caused by lack of CRH production [28]). In primary AI, iron accumulates directly in the adrenal glands, impairing cortisol production by damaging the adrenal cortex. This disrupts the body’s ability to respond to stress, including infections or physical exertion. Iron deposition in the hypothalamus or the pituitary gland can lead to secondary adrenal insufficiency by reducing ACTH secretion. Without adequate ACTH stimulation of any cause, the adrenal glands produce insufficient cortisol, further contributing to adrenal dysfunction. Patients with bThal and AI may experience few, if any, symptoms, which may be obscured by common symptoms of bThal such as fatigue, muscle weakness, or joint pain [29].

Although there were a few studies in which patients with bThal were assessed for AI using basal cortisol levels, in our analysis, we used data from studies which assessed AI with dynamic tests (tetracosactrin, GST, or ITT); dynamic testing is considered able to label patients with AI more accurately [30]. The tetracosactrin stimulation test involves the intravenous or intramuscular administration of a synthetic fragment of ACTH with full biological activity. Following that injection, serum cortisol levels are measured [31] (sensitivity is reported to reach 85%, with specificity at 96% [32]). In the GST, glucose levels temporarily increase and stimulate insulin secretion in subjects without diabetes. This insulin surge lowers glucose levels, triggering a counter-regulatory response, which includes an increase in cortisol [31] (sensitivity is reported to reach 56%, with specificity at 83% [33]). By administering an intravenous bolus of insulin to induce severe hypoglycemia, the ITT stimulates a counter-regulatory response that evaluates the entire HPA axis [32] (sensitivity was recently reported to reach 100%, with specificity at 87% [30]). The ITT is considered to be the gold standard for diagnosing all types of AI, especially those involving hypothalamic and pituitary dysfunction, where the tetracosactrin stimulation test might yield a false-negative result [34].

The findings of this meta-analysis suggest that patients with bThal have a higher prevalence of AI compared to the general population. Nevertheless, we noted that fewer cases of AI were noted in studies that used ITT compared to those that used the tetracosactrin test. These differences may be attributed to differences in the diagnostic characteristics of the tests (please see above).

Older individuals with bThal had higher rates of AI compared to younger ones. This may be the result of more accumulated years of transfusions, with concomitant iron deposition and subsequent effects on the HPA axis.

Interestingly, older (pre-2000) studies practically did not report AI. We can speculate that this may be due to the shift in norms of transfusion therapy, as well as the fact that adrenal insufficiency may not have been as frequently suspected or checked, especially in patients with hemoglobinopathies. Over the years, the treatment paradigm for homozygous beta thalassemia has evolved towards more intensive transfusion and chelation therapy [35,36,37,38,39,40]. This approach aims to maintain hemoglobin levels within a certain range to prevent complications such as growth retardation, organ damage, and bone deformities. However, more frequent transfusions can lead to iron overload and secondary hemochromatosis, which in its turn necessitates more aggressive chelation therapy to remove excess iron from the body. Side effects of intensive transfusion and chelation can include iron overload-related complications such as hepatic damage, cardiac problems, and endocrine dysfunction. Secondary hemochromatosis could affect and compromise all levels of the HPA axis. Balancing the benefits and risks of transfusions and chelation therapy is crucial in managing homozygous beta thalassemia effectively.

The limitations of the present study include the relatively small number of studies which were fit to be considered for inclusion, totaling a relatively small number of patients. Moreover, a few studies (or parts of them) were unfortunately left out of the analysis; in two of them, no details on the assessment/exclusion of AI were given [25,26], while in another (older) one, the authors reported that no cases of AI were found, using, however, only basal plasma cortisol measurements. In the latter study, the results were detailed graphically, and careful evaluation of the relevant figure shows almost all the basal cortisol measurements to be in the “grey” zone between 100 nmol/L and 400 nmol/L [24]. Additionally, we should point out the heightened heterogeneity of the results. Nevertheless, we believe that this does not invalidate our findings, since it is apparent that in studies after the year 2000, the results point unanimously to the direction of AI in approximately 30% of patients with bThal, with an increasing trend with advancing age, a finding that reflects the burden of repeated transfusions on the functionality of the HPA axis.

Current guidelines recommend assessment of the HPA axis’ integrity every 1–2 years in patients with bThalwho are being treated for growth hormone deficiency (since there is potential for concomitant anterior pituitary hormone deficiencies) [41]. In light of our findings, it may be prudent to consider glucocorticoid coverage in all patients with bThal who are undergoing surgical procedures, such as gall bladder surgery or splenectomy, to prevent potential adrenal crises. However, further research is needed to confirm these findings, to differentiate primary versus secondary AI, and to establish optimal strategies for the screening and management of AI in this patient population.

## 5. Conclusions

In conclusion, this meta-analysis underscores the higher prevalence of adrenal insufficiency among patients with bThal. The observed diagnostic variability emphasizes the need for standardized criteria for AI diagnosis in this patient cohort. Clinicians should be aware of the increased risk of AI in these patients and consider tailored management strategies to optimize patient care and outcomes. Future research should focus on elucidating the underlying mechanisms and refining diagnostic approaches for adrenal dysfunction in transfusion-dependent anemias.

## Figures and Tables

**Table 1:** Description of the studies which were included in the analysis: GST (glucagon stimulation testing); ITT (insulin tolerance testing).

Study	Subjects (*n*)	Subjects with Adrenal Insufficiency (*n*)	Testing Modality	Subjects
Hamidieh et al., 2018 [27]	21	3	Tetracosactrin	Young persons
Ucar et al., 2016 [7]	121	39	Tetracosactrin	Young persons
Jaruratanasirikul et al., 2007 [8]	48	22	Tetracosactrin	Young persons
Srivatsa et al., 2005 [9]	20	9	Tetracosactrin	Young persons
Karamfar et al., 2003 [10]	128	22	Tetracosactrin	Young persons
El Sedfyet al., 2011 [11]	45	7	Tetracosactrin	Young persons
McIntosh et al., 1973 [12]	9	0	Tetracosactrin	Young persons
McIntosh et al., 1976 [13]	9	2	Tetracosactrin	Young persons
Vannasaeng et al., 1981 [15]	7	0	Tetracosactrin	Young persons
Lassman et al., 1974 [16]	8	1	Tetracosactrin	All ages
Ambrogio et al., 2018 [17]	72	15	Tetracosactrin	Adults
Scacchi et al., 2010 [18]	56	18	Tetracosactrin	Adults
Baldini et al., 2017 [6]	3	3	Tetracosactrin	Adults
De Sanctis et al., 2016 [19]	3	3	Tetracosactrin	Adults
Huang et al., 2015 [20]	3	0	GST and ITT	Adults
Huang et al., 2015 [20]	8	1	GST and ITT	Young persons
Poomthavorn et al., 2010 [21]	26	21	ITT	Young persons
Kwan et al., 1995 [22]	68	1	ITT	Young persons
Sklar et al., 1987 [23]	8	0	ITT	Young persons
Total	663	167		

## Data Availability

No new data were created or analyzed in this study. Data sharing is not applicable to this article.

## Supplementary Materials

The following supporting information can be downloaded at https://www.mdpi.com/article/10.3390/medicina60101571/s1. Supplemental Table S1: Scoring of the included studies with the modified Newcastle–Ottawa scale University of Pennsylvania Center for evidence-based practice; Supplemental Figure S1: Funnel plots of the studies that were included in the analysis, which implemented testing with tetracosactrin (Supplemental Figure S1a; Q: 55.46; *p* < 0.0001; I^2^: 76.56%; 95% CI for I^2^: 60.81 to 85.98), assessment with insulin tolerance testing (Supplemental Figure S1b; Q: 73.36; *p* < 0.0001; I^2^: 94.55%; 95% CI for I^2^: 90.04 to 97.01), with young persons only (Supplemental Figure S1c; Q: 113.34; *p* < 0.0001; I^2^: 87.65%; 95% CI for I^2^:81.28 to 91.85), with adults only (Supplemental Figure S1d; Q: 20.14; *p*: 0.0002; I^2^: 85.11%; 95% CI for I^2^: 63.09 to 93.99), before the year 2000 (Supplemental Figure S1e; Q: 6.70; *p*: 0.24; I^2^: 25.48%; 95% CI for I^2^: 0.00 to 68.75), and after the year 2000 (Supplemental Figure S1f; Q: 78.84; *p* < 0.0001; I^2^: 84.78%; 95% CI for I^2^: 75.52 to 90.54); Supplemental Figure S2: Forrest plots of the studies that were included in the analysis, which implemented testing with tetracosactrin (Supplemental Figure S2a; percentage of adrenal insufficiency: 25.95% [fixed effects] and 27.24% [random effects]), assessment with insulin tolerance testing (Supplemental Figure S2b; percentage of adrenal insufficiency: 14.53% [fixed effects] and 17.16% [random effects]), with young persons only (Supplemental Figure S2c; percentage of adrenal insufficiency: 22.32% [fixed effects] and 20.33% [random effects]), with adults only (Supplemental Figure S2d; percentage of adrenal insufficiency: 29.99% [fixed effects] and 51.51% [random effects]), before the year 2000 (Supplemental Figure S2e; percentage of adrenal insufficiency: 4.15% [fixed effects] and 5.35% [random effects]), and after the year 2000 (Supplemental Figure S2f; percentage of adrenal insufficiency: 29.25% [fixed effects] and 35.36% [random effects]).
