# Supplementary material for: Adrenal Insufficiency in Patients with Beta Thalassemia: A Meta-Analysis
Source: Medicina (Kaunas). 2024 Sep 25;60(10):1571. doi: 10.3390/medicina60101571 (PMC11509364; doi:10.3390/medicina60101571)
Supplement: Supplementary file 1 [file medicina-60-01571-s001.zip › medicina-3204879-supplementary.pdf]

Supplemental Table S1

Modified Newcastle-Ottawa scale University of Pennsylvania Center For Evidence based Practice

Study population:

*All study groups derived from similar source/reference populations?

*Attrition not significantly different across study groups?

Study validity:

*The measure of exposure is valid?

*The measure of outcome is valid?

*Investigators blinded to endpoint assessment?

Confounders:

*Potential confounders identified (e.g., comorbidities, multicomponent interventions, etc.)?

*Statistical adjustment for potential confounders done?

*Funding source(s) disclosed and no obvious conflict of interest?

Reference: Wells G, Shea B, O'Connell J, Robertson J, Peterson V, Welch V, et al. The Newcastle-Ottawa scale (NOS) for assessing the quality of nonrandomized studies in meta-analysis. Available at http://www.ohri.ca/ programs/clinical_epidemiology/oxfordasp. Accessed June 21, 2016.

Study	Hamidieh 2018	Ambrogio 2018	Scacchi 2010	McIntosh 1973	Ucar 2016	Jaruratanasirikul 2007	Srivatsa 2005	Poomthavorn 2010	Huang 2015	Baldini 2017
[1]. Representativeness of the exposed cohort	1	1	1	1	1	1	1	1	1	1
[2]. Selection of the non-exposed cohort	0	0	0	0	0	0	0	0	0	0
[3]. Ascertainment of exposure	1	1	1	1	1	1	1	1	1	1
[4]. Precision of Exposure Dose Ascertainment	1	1	1	1	1	1	1	1	1	1
[5]. Ascertainment of exposure done prospectively or retrospectively	0	0	0	0	0	0	0	0	0	0
[6]. Demonstration that outcome of interest was not present at start of study, OR baseline assessment	0	0	0	0	0	0	0	0	0	0
[7]. Adjustment for confounding (rendering comparability of cohorts on the basis of the design or analysis)	0	0	0	0	0	0	0	0	0	0
[8]. Assessment of outcome	1	1	1	1	1	1	1	1	1	1
[9]. Was follow-up long enough for outcomes to occur?	0	0	0	0	0	0	0	0	0	0
[10]. Adequacy of follow-up of cohorts	0	0	0	0	0	0	0	0	0	0
Total	4	4	4	4	4	4	4	4	4	4

Study	De Sanctis 2016	Canale 1974	McIntosh 1976	Vannasaeng 1981	Lassman 1974	Karamifar 2003	Rafsanjani 2011	Elsedfy 2011	Torres 2002	Kwan 1995	Sklar 1987
[1]. Representativeness of the exposed cohort	1	1	1	1	1	1	1	1	1	1	1
[2]. Selection of the non-exposed cohort	0	0	0	0	0	0	0	0	0	0	0
[3]. Ascertainment of exposure	1	1	1	1	1	1	1	1	1	1	1
[4]. Precision of Exposure Dose Ascertainment	1	1	1	1	1	1	1	1	1	1	1
[5]. Ascertainment of exposure done prospectively or retrospectively	0	0	0	0	0	0	0	0	0	0	0
[6]. Demonstration that outcome of interest was not present at start of study, OR baseline assessment	0	0	0	0	0	0	0	0	0	0	0
[7]. Adjustment for confounding (rendering comparability of cohorts on the basis of the design or analysis)	0	0	0	0	0	0	0	0	0	0	0
[8]. Assessment of outcome	1	1	1	1	1	1	1	1	1	1	1
[9]. Was follow-up long enough for outcomes to occur?	0	0	0	0	0	0	0	0	0	0	0
[10]. Adequacy of follow-up of cohorts	0	0	0	0	0	0	0	0	0	0	0
Total	4	4	4	4	4	4	4	4	4	4	4

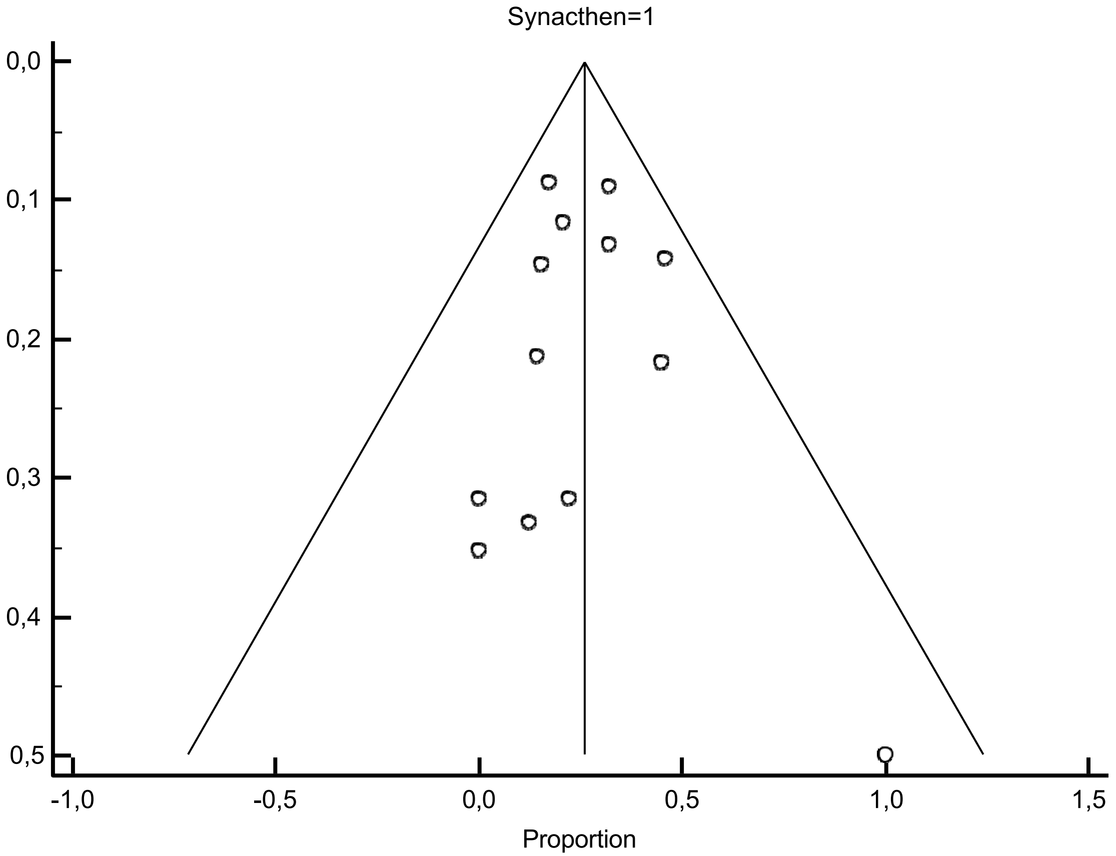

Supplemental Figure S1a

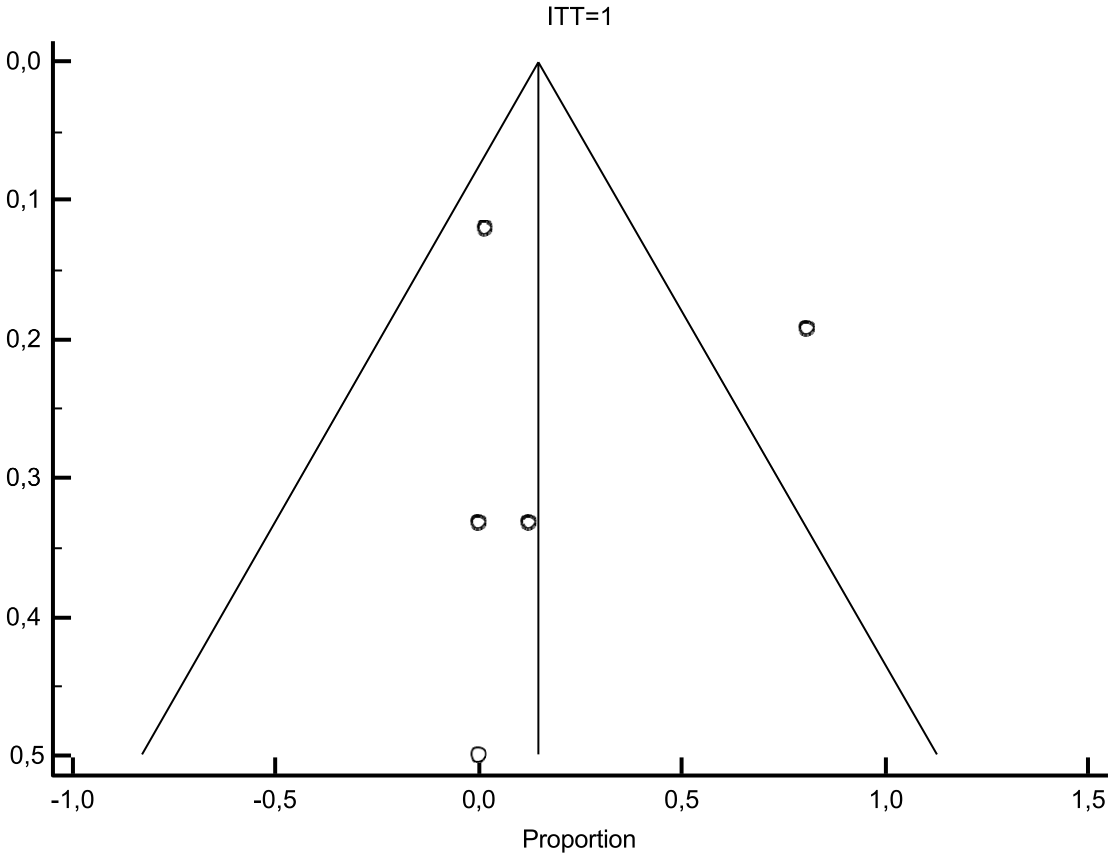

Supplemental Figure S1b

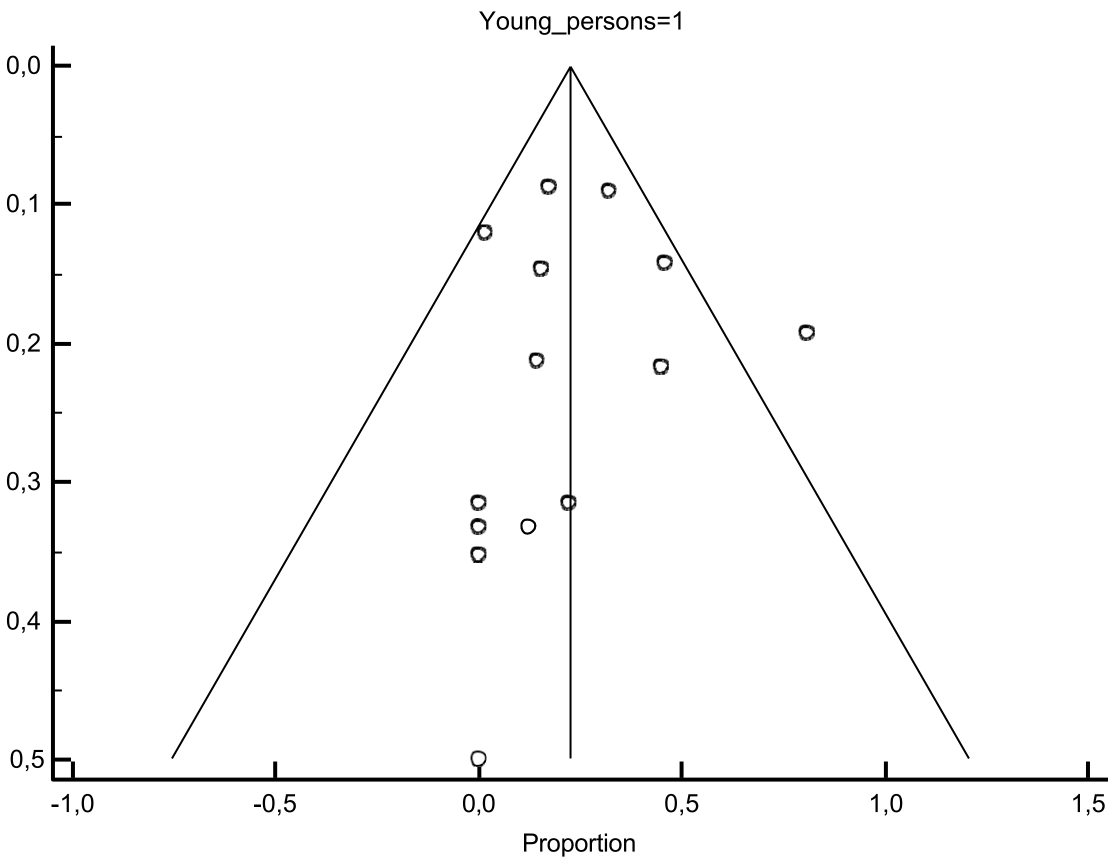

Supplemental Figure S1c

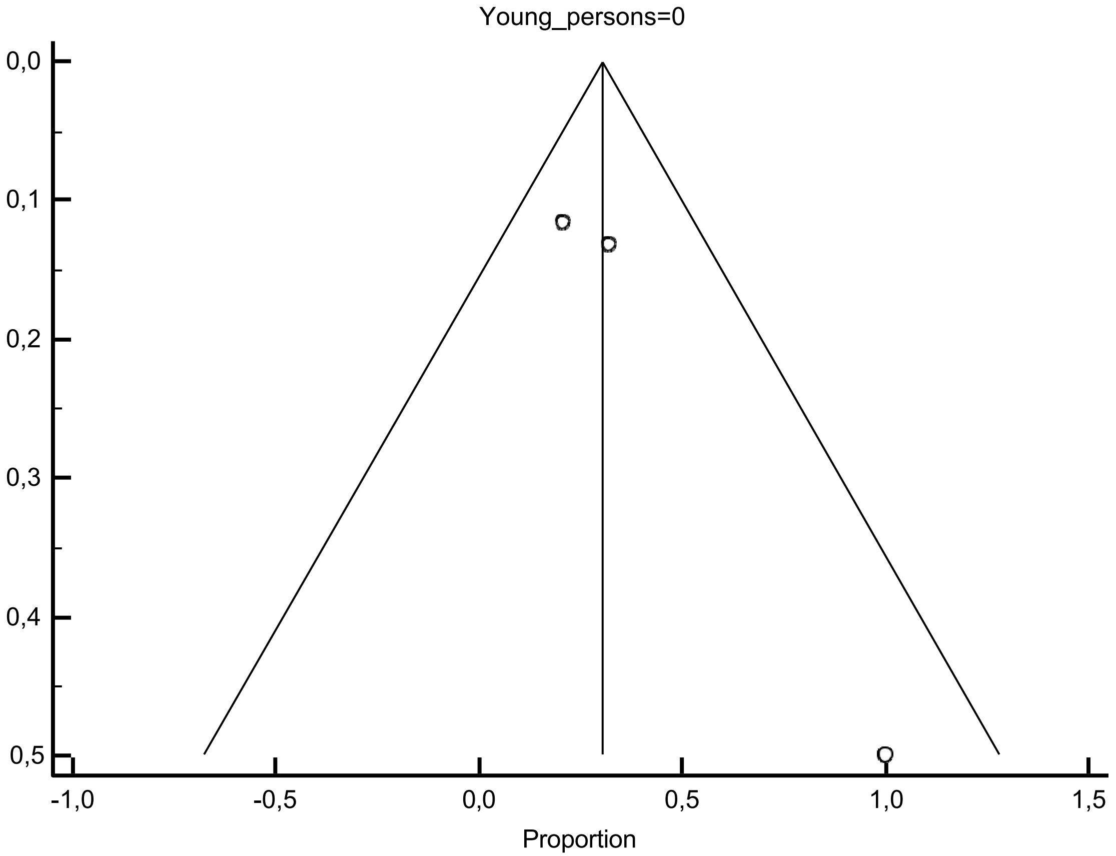

Supplemental Figure S1d

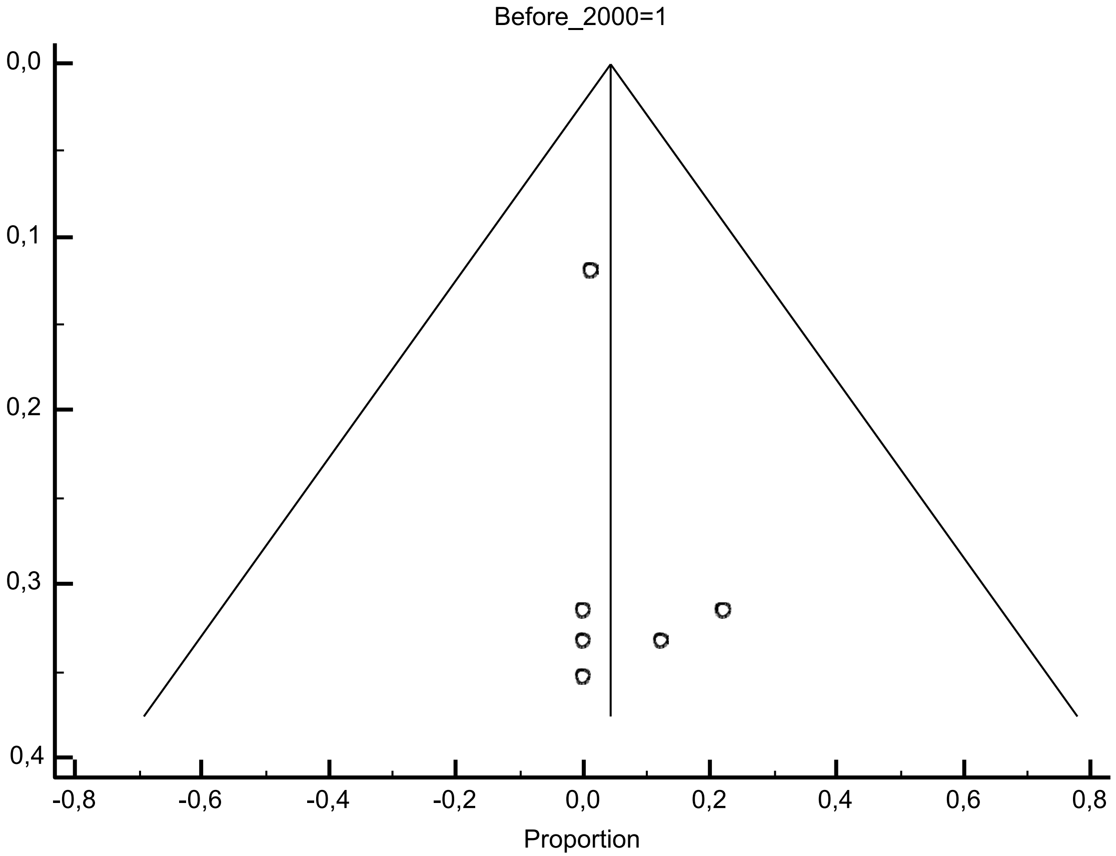

Supplemental Figure S1e

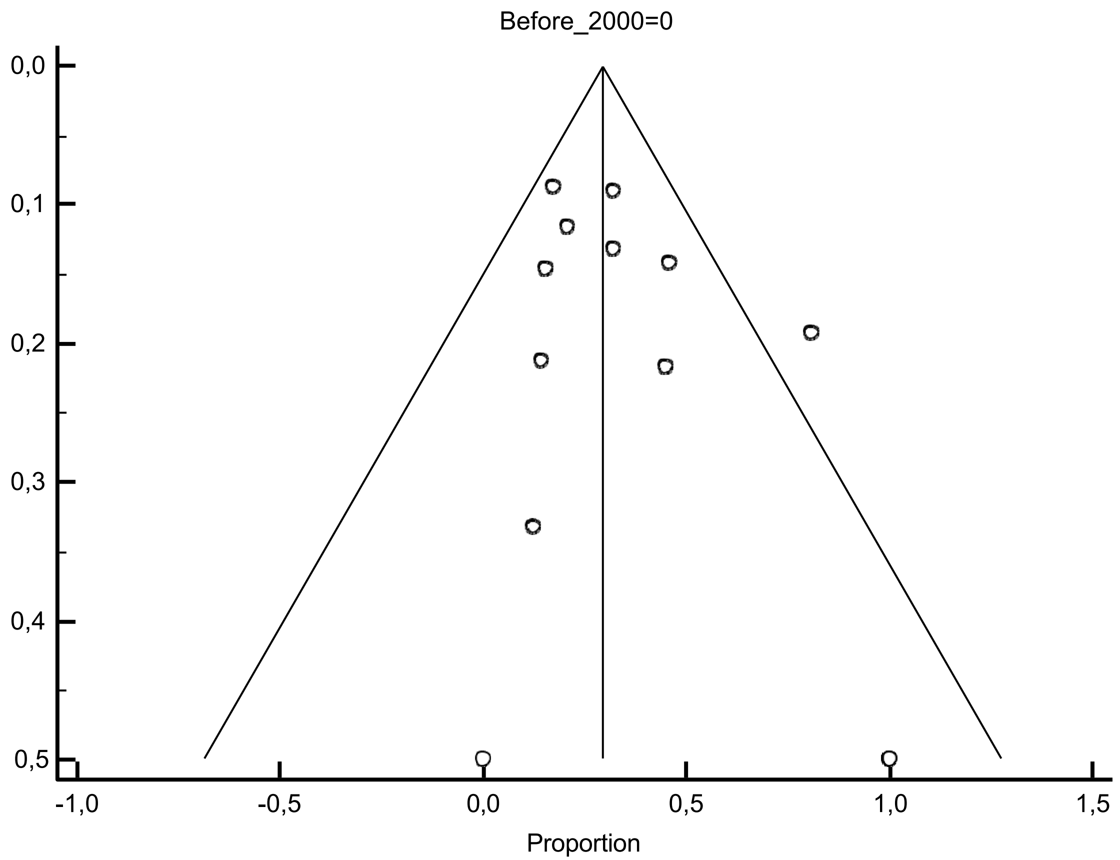

Supplemental Figure S1f

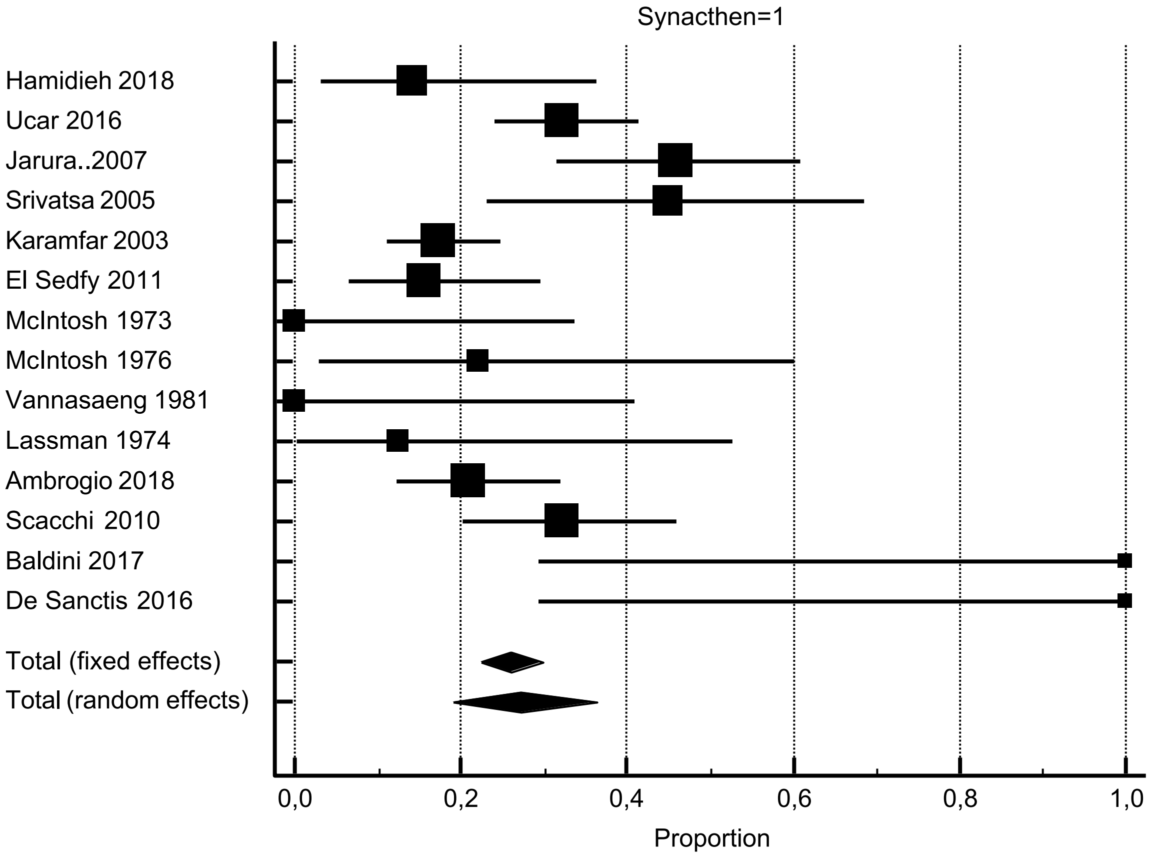

Supplemental Figure S2a

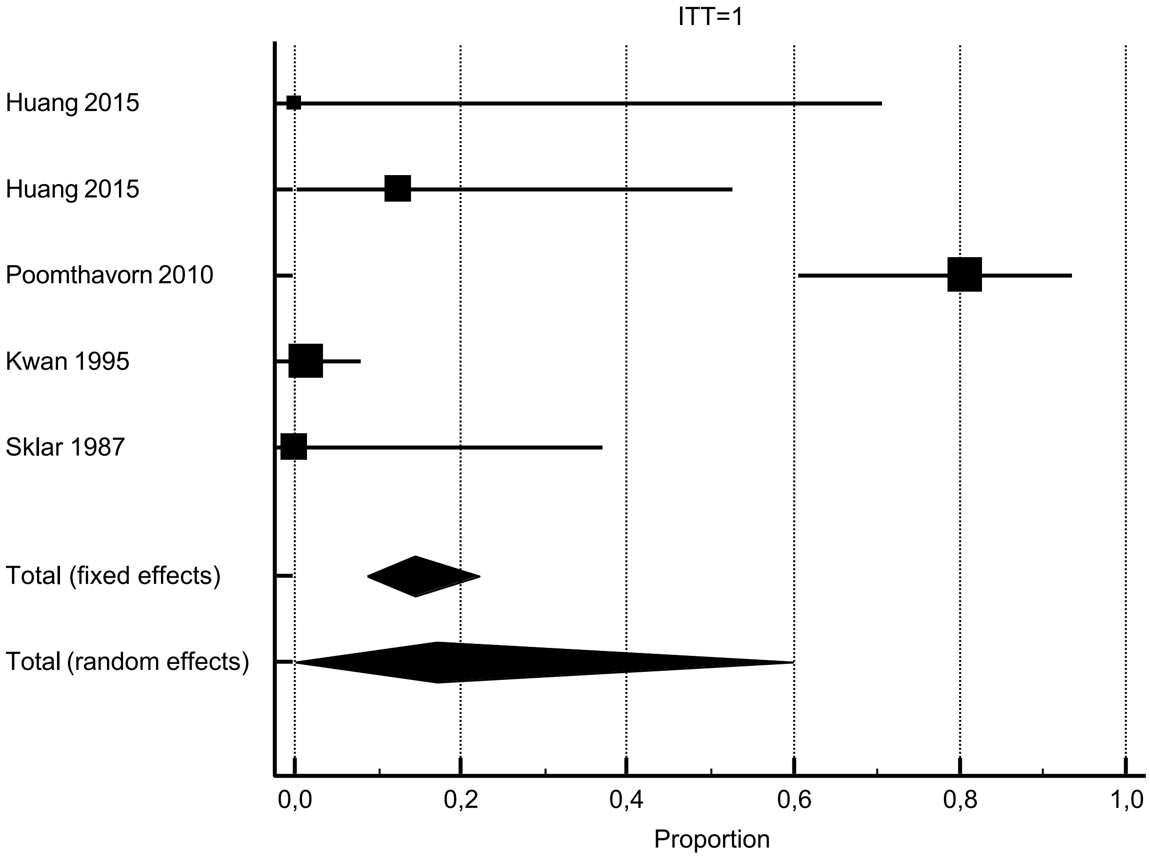

Supplemental Figure S2b

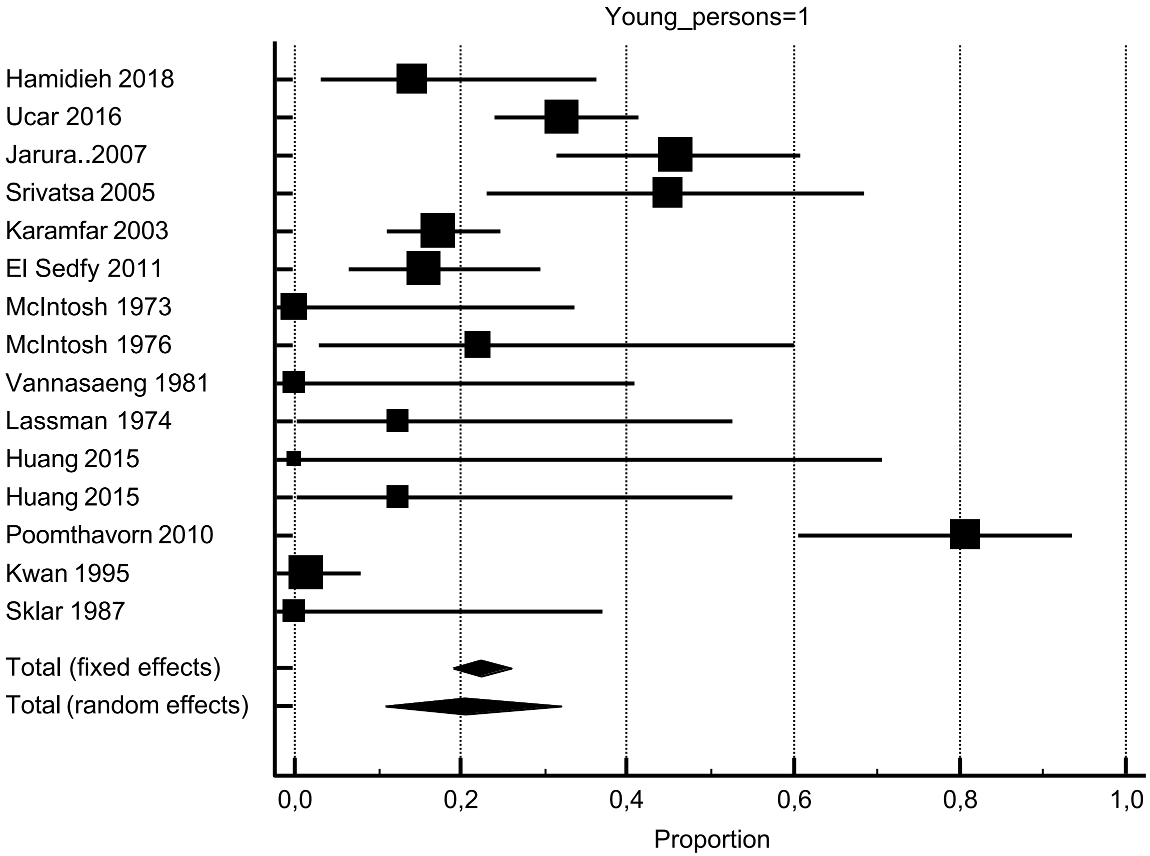

Supplemental Figure S2c

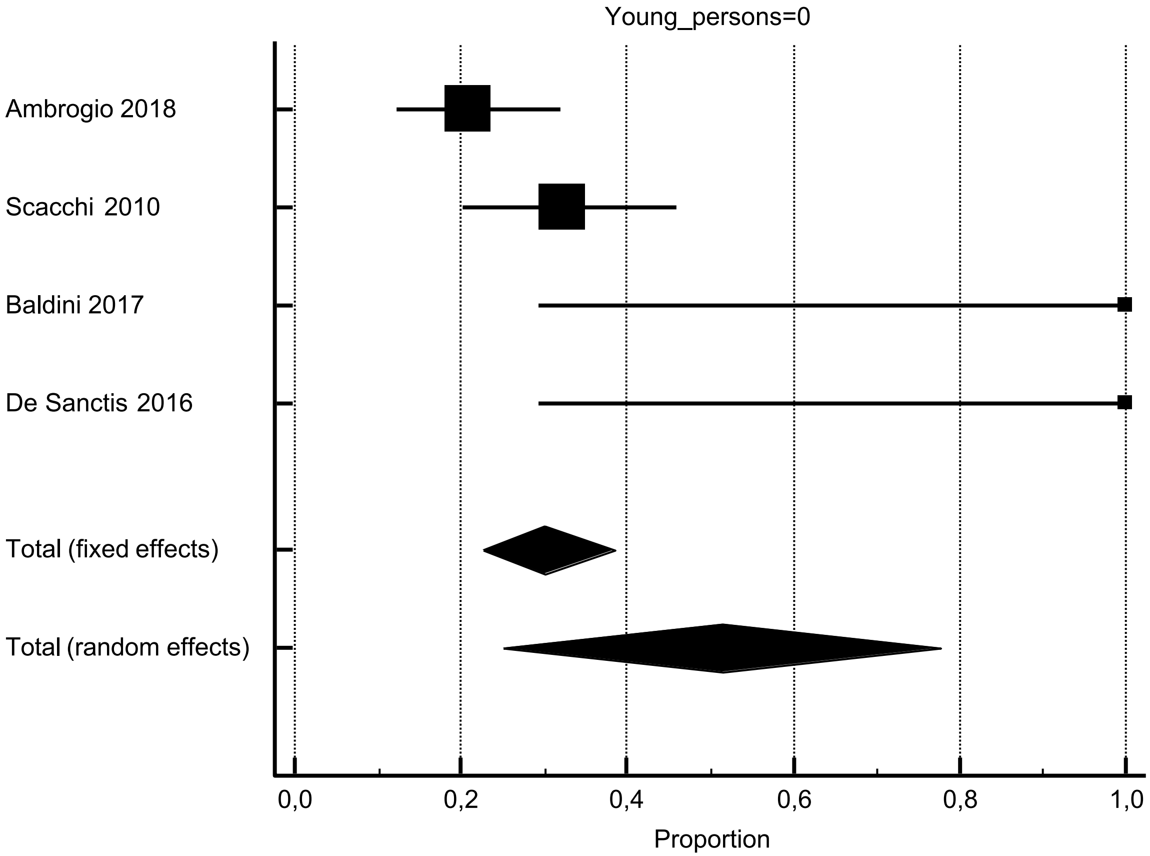

Supplemental Figure S2d

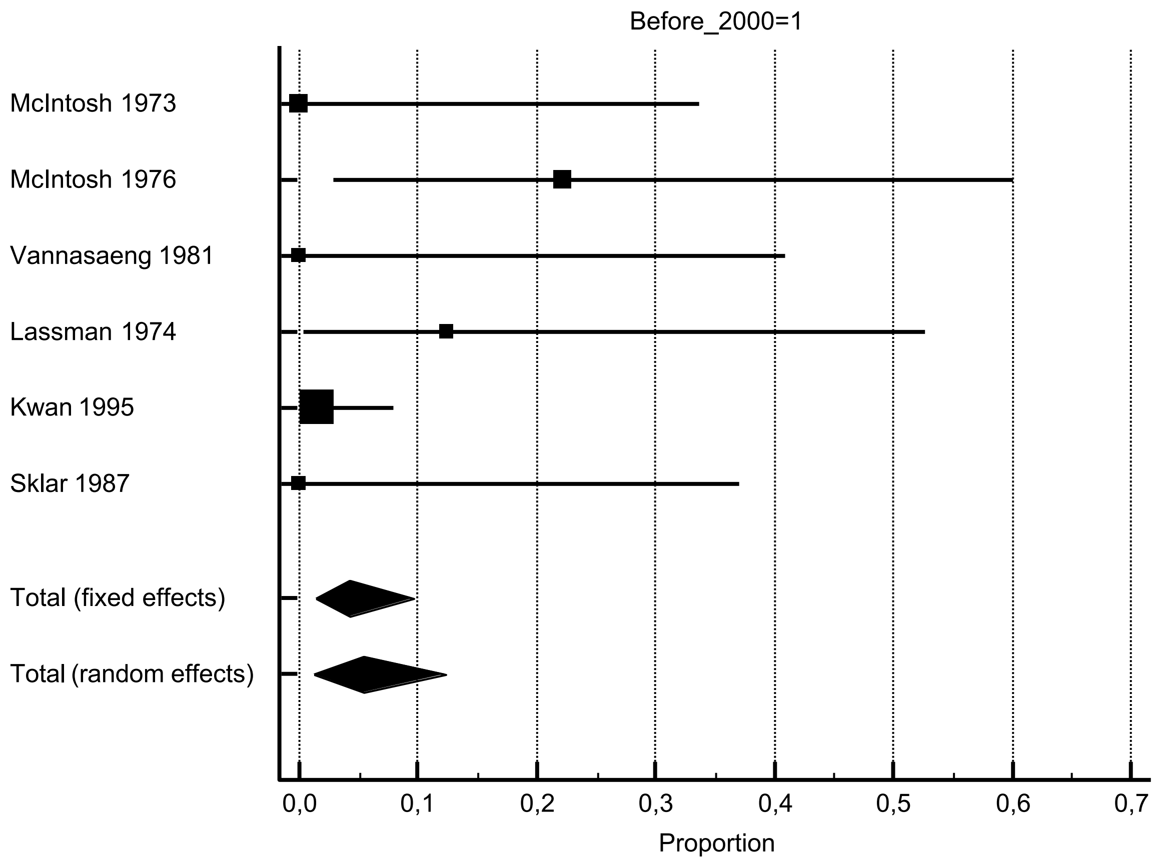

Supplemental Figure S2e

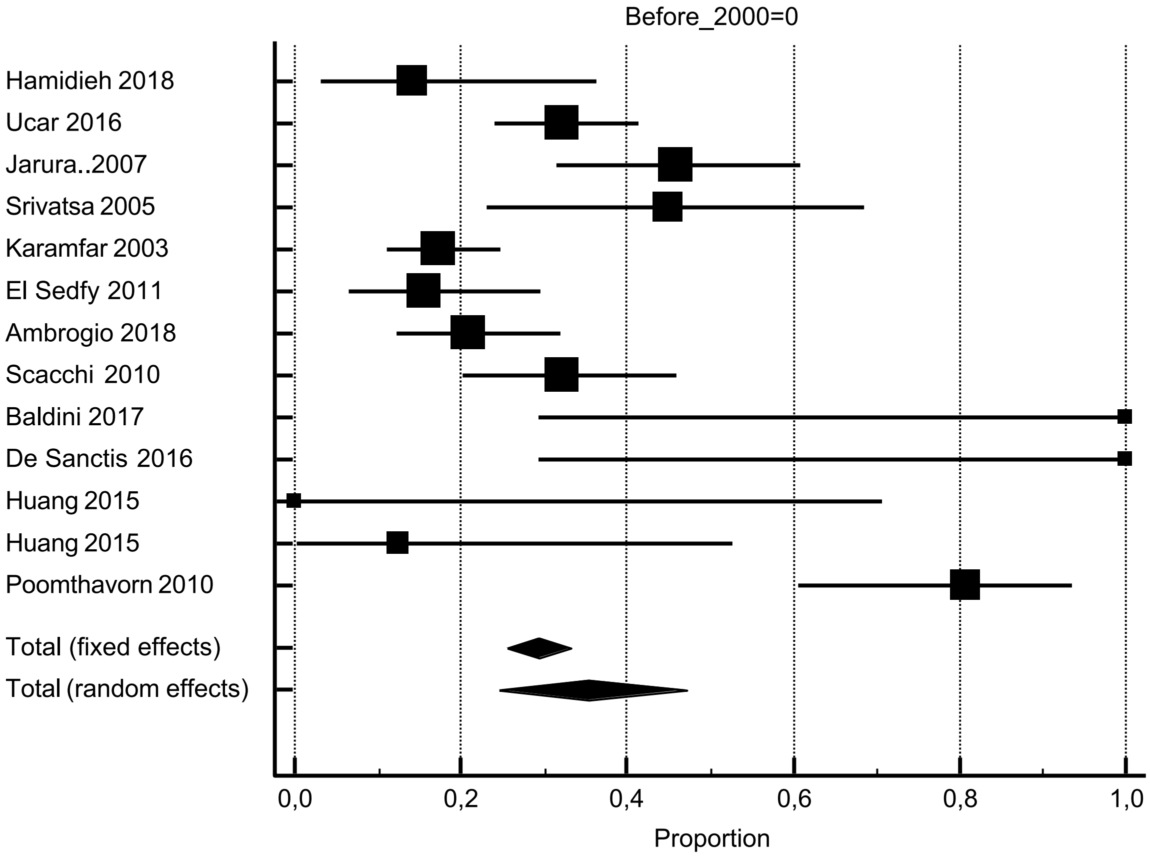

Supplemental Figure S2f
